# Neural response telemetry measures in patients implanted with Nucleus 24®

**DOI:** 10.1016/S1808-8694(15)31271-4

**Published:** 2015-10-20

**Authors:** Mariana Cardoso Guedes, Rubens V. Brito Neto, Maria Valéria S. Goffi Gomez, Sandra B. Giorgi Sant’Anna, Cristina G. Ornelas Peralta, Arthur Menino Castilho, Ricardo Ferreira Bento

**Affiliations:** 1 Speech and Hearing Therapist, Participant of the Cochlear Implant and Audiology Group, HC-FMUSP. Specialization in Audiology, Irmandade Santa Casa de Misericórdia de SP.; 2 Assistant Ph.D., Discipline of Otorhinolaryngology, HC-FMUSP.; 3 Speech and Hearing Therapist, Ph.D. in Sciences, Human Communication Disorders, UNIFESP.; 4 Speech and Hearing Therapist, Fundação Otorrinolaringologia. Master in Experimental Pathophysiology, FMUSP.; 5 Speech and Hearing Therapist, HC-FMUSP. Participant of the Cochlear Implant and Audiology Group, Master in Speech and Hearing Therapy, PUC-SP.; 6 Assistant Physician, HC-FMUSP. Intern, specialized course on otology and skull base surgery, HC-FMUSP.; 7 Associate Professor, Discipline of ORL, FMUSP.

**Keywords:** cochlear implant, compound action potential, electrical stimulation, objective measure

## Abstract

Cochlear implantation has been recommended for children under 24 months of age. The use of objective measures is needed to help speech processor programming. The electrically evoked compound potential (EAP), which can be assessed by neural response telemetry (NRT), is one of those objective measures. **Aim:** to determine how often the EAP can be recorded by NRT system during surgery and to describe the responses. **Study design:** clinical with transversal cohort. **Material and Method:** the impedances and NRT were measured in a group of 17 *Nucleus 24*^®^ implant users. The responses were analyzed and compared to the etiology, hearing loss duration and electrode array position. **Results:** The EAP was easily recorded in the apical electrodes and, in otosclerosis and meningitis cases the EAP threshold was higher than in the other etiology cases. **Conclusions:** The NRT can be found in 82% of the cases during surgery. The responses obtained may vary according to etiology and the position of electrodes along the cochlea.

## INTRODUCTION

Cochlear implant is the treatment of choice for bilateral sensorineural severe and profound hearing loss ^1^ and it requires programming of each electrode so that we reach appropriate levels of electrical stimulation. The programming is made in the speech processor of the patients, which determines how sound is going to be analyzed and codified by a strategy of speech codification ^2^. The stimulation unit used to program electrodes is arbitrary and it is named current unit (CU) and ranges from 1 to 255, corresponding to approximately 0.01mA and 1.75mA, respectively. One of the main challenges in the case of implants in small children is postoperative follow up, especially in the definition of current levels for stimulation that will determine the programming of the speech processor ^3^.

The success of the patient and satisfaction with the implant are highly dependent on the appropriateness of the speech processor programming, because it is the map that determines the sound and which characteristics are codified. However, in small children, this type of procedure requires attention and takes time, given that they are not capable of voluntarily specifying the auditory sensation generated by electrical stimulation, which may be tiring for the children and for the family alike. In such cases, behavioral thresholds are limited and responses can be inconsistent, reflecting levels of perception and discomfort that are not appropriate. Difficulties can be even greater in children with congenital hearing loss and in patients with multiple deficits, owing to lack of knowledge and familiarity with sound patterns, reduced attention span and no linguistic or cognitive skills required for such a task ^4^.

Thus, objective measures have been studied and employed to predict the levels of stimulation for the construction of the first maps and also for the verification of the integrity of the whole system. It is natural that these levels suffer small changes throughout time, and they should be complemented and adjusted with psychoacoustic measurements, as there is an increase in the subjects’ skills to detect, recognize and consistently respond to the sound stimulus ^3^.

There are many media to reach objective measures of the auditory nerve in users of cochlear implant from the electrical stimulation of the auditory system, such as evoked auditory brainstem potentials (ABR), middle latency responses and late potentials (P300 and Mismatch Negativity) and Stapedial Reflex study, many of them used to monitor the progression of the patients and in an attempt to define successful prognosis to the subject 5, 6, 7, 8.

The measurement of auditory nerve activity more frequently used in users of cochlear implant is EAP, or Evoked Action Potential. EAP is typically formed by a negative peak (N1) with approximate latency of 0.2ms to 0.4ms, followed by a positive peak (P1). The amplitude of response (measured between N1 and P2) ranges depending on increase of intensity of the stimulus and it is mediated in µV.

EAP thresholds may be useful to predict minimum and maximum levels that should be used in mapping electrodes for programming of speech processor, facilitating this process in children and determining some parameters for the stimulation that will result in programming of more appropriate levels, which may improve the performance of subjects 6, 9. EAP thresholds may be used to estimate electrical levels, but they cannot predict the exact values of psychophysiological measurements.

EAP is easily recorded owing to the characteristics of the multichannel cochlear implant *Nucleus 24*^®^ (CI24) and the existence of a specific software (system of telemetry). Telemetry is a mechanism that captures distant events, in which the first system can be used to measure impedance of each electrode, monitoring the appropriateness of electrical current generators. The second system, NRT Neural Response Telemetry is the method that enables capture of the action potential of the distal portion of the auditory nerve in patients users of implant CI24, using the implant itself to generate stimuli and to record the responses.

A series of intracochlear electrodes of CI24 comprises 22 electrode bands, numbered from 1 to 22, and 22 is the most apical one. In addition to these intracochlear electrodes, CI24 presents two extracochlear electrodes (MP1 and MP2) that allow monopolar stimulation, enabling MP1 electrodes to form a pair to generate stimulus, and another MP2, to form a pair to record responses in the auditory nerve^10^.

Impedance is related with resistance characteristic of fluid and tissue that involves the electrode chain and it is one factor that determines the consumption of energy from the cochlear implant system 11, 12. Telemetry of impedances should be performed before the telemetry of neural responses to confirm the functioning of receptor and stimulator and to check the existence of an open circuit and short circuit in the intracochlear electrodes from the measurement of their electrical resistance^10^.

The use of telemetry system is recommended in the intraoperative condition so as to check not only the integrity of the electrode chain after the cochlear insertion, but also because sedation allows the use of currents with greater intensities without causing patients’ discomfort, increasing the chance of capturing responses ^7^. NRT values found in the intraoperative condition could be used to build the first maps^3^.

Significant correlations between the level of response obtained with NRT and levels obtained by behavioral method have been studied, to use data of objective measures for the programming and mapping of electrodes^4^, ^8^, ^9^, ^13^, ^14^, ^15^.

Thus, EAP thresholds could be used in cases of patients that do not precisely determine the minimum levels of response (T levels) using behavioral methods, because they represent the intensity that is above the threshold. Therefore, upon defining T levels close to values obtained in EAP we would ensure that stimuli are audible and comfortable ^8^. Moreover, the value of inclination of the growth curve of amplitude (function I/O) may provide data about the dynamic field of the patients. The advantage to use objective measures in the construction of the map is the fact that it is required for final adjustment of the program to have only measurements of loudness perception (intensity sensation), whereas in the traditional method the patients have to judge 44 measures, which makes it very tiring and may interfere in their performance^14^, ^15^.

The purpose of the present study is to describe and characterize the intraoperative responses of the auditory nerve action potential obtained with the method of neural response telemetry (NRT), as well as prevalence in users of cochlear implant *Nucleus 24K*^®^.

## MATERIAL AND METHOD

### Method

The study included 54 users of cochlear implant *Nucleus*^®^ (*Cochlear Corporation*), successfully implanted by the Group of Cochlear Implant, Discipline of Otorhinolaryngology, Clinical Division of Ophthalmology and Otorhinolaryngology, Hospital das Clínicas, Medical School, University of Sao Paulo (HCFMUSP) between January and December 2003.

Out of 56 studied subjects, 29 were female and 25 were male, aged between 2 and 63 years (mean age of 26 years). Duration of deafness ranged from 1 to 35 years (mean of 8.7 years), and 48% of subjects presented deafness for at least 5 years; 28% presented it from 5 to 10 years; 13%, from 10 to 15 years, and 11% presented deafness for more than 15 years. As to etiology, meningitis was determining in 30% of the cases; congenital loss in 31%, progressive deafness (cochlear otosclerosis, hereditary hearing loss and idiopathic cases) was seen in 26% of the cases and sudden deafness in 7% of the cases (Table 1).

**Table 1:** Characterization of subjects.

PATIENT	GENDER	AGE	ETIOLOGY	DURATION OF DEAFNESS (IN YEARS)	IMPLANT	INSERTION
1	M	17	Usher syndrome	17	CI24k	Complete
2	M	39	Meningitis	30	CI 11+11+2	Complete
3	F	9	Meningitis	9	CI24k	Complete
4	M	11	congenital	11	CI24k	Complete
5	M	40	Otosclerosis	14	CI24k	Complete
6	F	5	congenital (maternal rubella)	5	CI24k	Complete
7	M	8	Meningitis	6	CI24k	Partial
8	F	23	progressive (genetic)	2	CI24k	Complete
9	M	42	progressive	10	CI24k	Complete
10	M	12	Meningitis	1	CI 11+11+2	Partial
11	M	30	Ototoxicity	5	CI24k	Complete
12	F	30	Meningitis	14	CI24k	Complete
13	F	42	Meniere	4	CI24k	Complete
14	M	9	Congenital	9	CI24R (CS)	Complete
15	F	2	Meningitis	2	CI 11+11+2	Partial
16	M	5	congenital (CMV)	5	CI24k	Complete
17	F	4	congenital (genetic)	4	CI24k	Complete
18	F	3	congenital	3	CI24R (CS)	Complete
19	F	2	congenital (leukodystrophy)	2	CI24k	Complete
20	F	23	Progressive	2	CI24k	Complete
21	M	6	Meningitis	6	CI 11+11+2	Complete
22	F	6	congenital (prematurity)	6	CI24k	Complete
23	M	5	congenital (genetic)	5	CI24k	Complete
24	M	30	Meningitis	10	CI24k	Complete
25	F	45	Meningitis	5	CI24k	Partial
26	F	30	Meningitis	15	CI24k	Complete
27	M	5	congenital (genetic)	5	CI24k	Complete
28	M	35	Meningitis	15	CI 11+11+2	Complete
29	M	22	Meningitis	22	CI24k	Complete
30	F	40	Meningitis	35	CI24k	Complete
31	F	43	Progressive	2	CI24k	Complete
32	M	50	Progressive	5	CI24k	Complete
33	M	50	Meningitis	30	CI24R (CS)	Complete
34	F	60	Progressive	2	CI24R (CS)	Complete
35	F	43	Progressive	7	CI24k	Complete
36	F	60	Progressive	3	CI24k	Complete
37	M	38	Otosclerosis	2	CI24R (CS)	Complete
38	F	60	progressive (genetic)	5	CI24k	Complete
39	M	5	congenital (genetic)	5	CI24k	Complete
40	F	29	Head trauma	10	CI24k	Complete
41	F	60	Otosclerosis	10	CI24k	Partial
42	F	12	Meningitis	11	CI24k	Complete
43	M	55	head trauma	7	CI24k	Complete
44	M	8	congenital	8	CI24k	Complete
45	F	17	enlarged vestibular aqueduct syndrome	2	CI24k	Complete
46	M	30	Meningitis	30	CI24k	Complete
47	M	35	Progressive	2	CI24k	Complete
48	M	60	Progressive	5	CI24k	Complete
49	F	43	Progressive	2	CI24k	Complete
50	F	63	Progressive	14	CI24k	Complete
51	F	7	congenital (jaundice)	7	CI24k	Complete
52	F	9	congenital (genetic)	9	CI24k	Complete
53	F	4	congenital (prematurity)	4	CI24k	Complete
54	F	10	congenital (maternal rubella)	10	CI24k	Complete

F: female / M: male / CMV: cytomegalovirus / CI24k: model *nucleus 24 kids* / CI24R(CS): model *nucleus 24 contour* / CI 11+11+2: model *nucleus 24 double array*.

### Procedure

EAP was performed with software NRT 3.0, installed in a portable microcomputer coupled to the interface of portable programming system PPS and speech processor model SPrint^®^, both produced by Cochlear Corporation.

During surgery, right after the insertion of electrodes in the cochlea, when the patient was anesthetized, we performed the assessment of the cochlear implant integrity (impedance telemetry). Impedance telemetry was performed at 250 pulses per second current for 25µs per phase and mean intensity of 100 current units (equivalent to approximately 0.2mA). Impedances were measures in modes monopolar MP1, monopolar MP2, monopolar MP1+2 and Common Ground (CG). Values were considered normal when between 1.5 kilo ohms (kΩ) and 20kΩ in modes MP1, MP2 and MP1+2 and between 0.7kΩ and 20kΩ in mode CG. Electrodes with abnormal impedances were not used for NRT recording, being replaced by an adjacent electrode.

Measurement of NRT, performed next, used the paradigm of anticipated masking and subtraction method, in which the stimulus is transmitted with specific delay in relation to masking (data in sequence) to use the period refractory to auditory pathway neurons. The response to stimulus in such conditions is subtracted from responses to isolated stimuli, in an attempt to eliminate the artifacts and enable observation of the potential. The subtraction method is used automatically by the software to separate the neural responses from electrical artifacts. The masker level was placed at 10 current units above the level used to stimulation or probe level. The interpulse interval was fixed at 500 µs and the stimulation speed was at 80Hz in series of 25µs per phase. The other parameters, such as amplifier gain, delay (interval between end of stimulus and response recording) and distance between MP1 and MP2, were adjusted and modified according to what was proposed by Abbas et al. (1999)^6^ to reach the best wave morphology. The suggested gain by the manufacturer is of 60dB, but it was adjusted for 40dB when the responses presented saturation. The delay was defined according to number of artifacts so as to enable better visualization of wave N1. The number of stimulus presentations ranged from 100 to 200 for gains of 60dB and 40dB, respectively.

The amplitude growth curve was obtained by investigating 4 to 7 waves with different intensities, presenting an interval of five current units each, for tested electrodes (electrodes 20, 15, 10, 5 and 3). Latencies in peaks N1 and P1 were manually measured. Amplitude of EAP was determined by the difference in voltage between waves N1 and P1, and the program applied an adjustment in linear regression about the curve of amplitude growth, determining the thresholds of EAP and the inclination of the curve (I/O function) for each studied electrode.

For data analysis, subjects were divided into groups according to the etiology of deafness in the implanted ear and according to the duration of deafness. Electrodes were divided according to their position in the cochlear in apical electrodes (22 to 16), medial (15 to 8) and basal (7 to 1).

## RESULTS

To reach the appropriate morphology for EAP, obtained by NRT system, the amplifier gain required reduction of 30.6% for the investigated electrodes, whereas the other 63.9% had only the normal delay. We tested 227 electrodes.

NRT responses were obtained in 80% of the assessed patients and in 75% of the tested electrodes, and the prevalence was higher in apical electrodes (76%) in relation to medial ones (74%) and basal ones (74%). Only 10 subjects did not present responses in any of the tested electrodes, and out of the total, 60% presented etiology of meningitis.

The number of patients with affections to impedance telemetry test was 23% (7 subjects). The electrodes with abnormal impedance were not used for measurement of potential.

**Graph 1:**
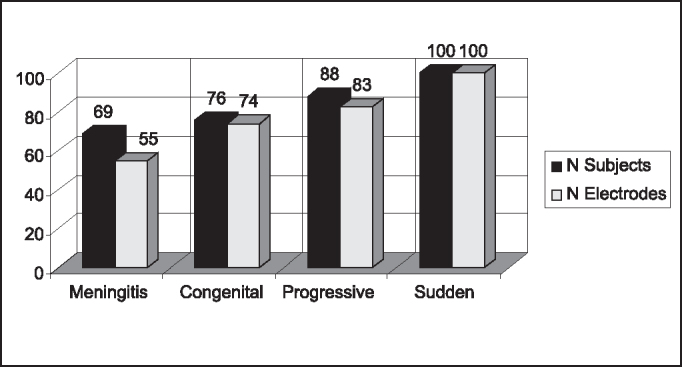
Prevalence of NRT (%) based on deafness etiology.

**Graph 2:**
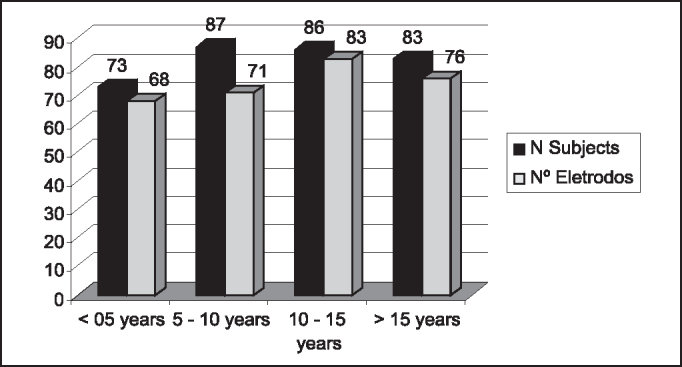
Prevalence of NRT (%) based on duration of deafness.

As to prevalence of potential, Graphs 1 and 2 show these data grouped by etiology and duration of deafness, by subject and tested electrode.

The main variation of thresholds in the same subject was 10CU. The mean threshold of the group was 190 CU (standard deviation of 12.92).

The mean inclination of amplitude growth curve (I/O function) was 9.79 (standard deviation of 6.92). The mean intra-subject variability was 4.4; however, never higher than that between electrodes placed on the same region of the cochlea.

Table 2 describes the thresholds obtained for NRT testing and the value of I/O function of amplitude growth curve with etiology. Table 3 makes the same description, but according to duration of deafness.

**Table 2:** NRT thresholds and value of I/O function according to deafness etiology.

ETIOLOGY	pNRT (mean in CU)	I/O function
Meningitis	192	9.39
Congenital Deafness	189	10.9
Progressive Deafness	189	8.99

Others (sudden causes)	188	10.21

CU: current unit

**Table 3:** NRT thresholds and value of I/O function according to duration of deafness.

Duration of deafness	pNRT (mean in CU)	I/O Function
Up to 5 years	189	11.8
Between 5 and 10 years	192	8.5
Between 10 and 15 years	192	8.9
Over 15 years	188	8.6

CU: current unit

Latency N1 was found between 0.27µs and 0.41µs in all subjects (mean of 0.33µs) and it did not vary more than 0.09µs in the same patient. As to duration of deafness, the mean latency was the same in all groups (0.34µs). According to the presented etiologies, the mean latency ranged between 0.32µs (ototoxicity, otosclerosis and idiopathic deafness) and 0.34µs (congenital deafness by head trauma and meningitis).

Characteristics of action potential of 8^th^ cranial nerve obtained by NRT were also analyzed according to position of the electrode on the cochlea, as shown in Table 4.

**Table 4:** Characteristics of NRT (thresholds, I/O function and latency) according to the position of the electrode in the cochlea.

	Apical	Medial	Basal	Total
Thresholds (mean in CU)	187	190	193	190
Latency (mean in ms)	0.32	0.34	0.34	0.33
I/O function (mean)	10.44	9.29	10.06	9.79
Prevalence (%)	76	74	74	75

CU: current unit

## DISCUSSION

NRT may be easy and quick to perform, can be used during surgery to measure responses of auditory nerve peripheral portion from electrical stimulation and also to check the integrity of the electrode chain when it is inserted in the cochlea ^7^. In the present study, EAP was satisfactorily obtained with the use of the telemetry system during the surgeries of cochlear implant, especially because it is a relatively quick method that does not affect the surgeon’s schedule.

EAP presents advantages relative to ABR, especially because it does not need external electrodes placed on the head surface, it is less susceptible to myogenic interference and needs a smaller number of stimuli to be triggered. Given that they are measured directly from intracochlear electrodes, EAP amplitude tends to be greater than that of other potentials 3, 8, 9. Moreover, the use of this measurement may help in the selection and exclusion of specific electrodes during the programming of speech processor ^6^.

Potential morphology, latencies and amplitude growth curve inclination (I/O function) observed in the present study were similar to those described in other studies 6, 8, 9, 14.

The prevalence of responses was of 75% of electrodes, obtained in 80% of the subjects and slightly higher in apical electrodes, followed by medial and basal electrodes. The higher prevalence was in apical electrodes and it may related with auditory residue, which is more present in the cochlear region^6^, ^14^, ^15^.

Impedance affections were found in 13% of the subjects that had electrodes with values over 20kΩ (N=8) or below 1.5 kΩ. Similar data were previously reported in which impedance affections were observed in 16%^8^, 20%^9^ and 23%^11^ of the subjects. High values of impedance may suggest, for example, air bubbles inside the cochlea, electrodes outside the cochlea (partial insertion), electrodes in the epitympanic space or electrodes in the open circuit. Lowered values in turn, may suggest cochlear or common cavity malformations; electrodes touching (double chain), excess of saline solution in the mastoid cavity, or electrodes in short circuit. The advantage of this procedure in the intraoperative moment is that depending on the alteration, the surgeon should try to solve the problem before closing the cavity, repositioning the electrodes, for example, or deciding to replace them in case the number of electrodes with affected impedance is high.

They describe affections of impedance seen in patients without surgical complications and with pervious cochlea ^11^. All patients that presented impedance affections in more than five electrodes (N=7) did not present responses in neural telemetry, even when using other electrodes for simulation and recording of potential. In x-ray exams (intraoperative) we evidenced the partial insertion of the cochlea implant. We can assume that the prevalence of intraoperative responses is somewhat related with affections of impedance and conditions of electrode insertion. In such cases, 71% of them presented meningitis as etiology, with partial or total cochlear ossification. To program the speech processor, electrodes with affected impedance were disabled.

Smoorenburg et al.^13^ did not manage to reach NRT responses in 14 out of 27 subjects. As the procedure was performed in a post-surgical session, authors attributed it to low prevalence at the level of discomfort of these patients that did not enable the increase in current units necessary to collect the potential. In the other patients (N=13), they found satisfactory responses in 20 electrodes. In our sample, the prevalence was higher in part owing to the fact that the patients were anesthetized, enabling potential investigation at high current intensity without causing discomfort 3, 7.

Patients that did not present responses in intraoperative conditions should be reassessed to confirm the results during the first stimulations, because absence of potential may have been caused by lack of appropriateness of stimulation parameters, given that during surgery sometimes there is not enough time for many modifications or more detailed affections. It is recommended to further investigate with electrically evoked stapedial reflex. The association of telemetry responses and stapedial reflex is extremely important in programming cochlear implants, especially when determining the comfort levels^7^.

The prevalence of responses was markedly smaller, by number of patients and tested electrodes, in cases of meningitis. All patients in which responses were absent presented some degree of cochlear ossification. Moreover, it is known that meningitis may cause death of a large number of ganglionar cells and even neural tissue damage ^5^. Gantz et al.^5^ also found increase in neural recovery in cases of meningitis during NRT study. Moreover, they observed that children with post-meningitis deafness took longer to progress in speech perception than those with congenital deafness.

A smaller prevalence of NRT responses in patients that had had deafness for up to 5 years was detected, even though not expected. However, it was the group with more patients that had had meningitis and partial insertion of electrodes, which might have influenced the results. If we exclude the cases in which electrode beam insertion was not complete, the group with deafness for 5 years would have had the higher prevalence (88%).

There was tendency of elevated thresholds obtained by NRT from basal electrodes. This finding was in agreement with previous studies 3, 9, 13 that in addition to more elevated thresholds in basal electrodes, also had amplitude of response reduced in the same electrodes. It is believed that the responses to electrical stimulation of electrodes could ranged according to the position in the cochlea and according to the density and integrity of neural population ^5^. Thus, we can infer that there is a correlation between residual hearing in low frequencies, which would result in smaller electrical thresholds in apical electrodes owing to higher concentration of functional neurons in this region of the cochlea. Conversely, the need to have higher intensity in the basal electrodes may also be related with the position in the tympanic membrane and the probable surgical damage on the base. It is known that the radial distance between the electrode chain and the modiolus is correlated with levels of energy for most users of cochlear implant^12^.

Mean of thresholds obtained with NRT in the present study (190CU) was close to that described in the literature (about 187CU and 185CU), as well as the variability between thresholds in the same subject 8, 9.

The investigation of objective responses of the auditory nerve through electrical stimulation in users of CI24 implant show little variability between adjacent electrodes, with slight increase of thresholds in basal electrodes, following the configuration of the map obtained through the behavioral method. Thus, we developed a formula to predict the levels of stimulation of all electrodes based on objective responses of NRT associated with behavioral responses for one single electrode. In general, thresholds obtained with NRT represent about 61% of the dynamic field in children users of speech codification strategy SPEAK^®^. As shown in the studies, NRT measures may be used in the production of electrical levels to be applied in smaller children 3, 9. In adult patients, EAP thresholds tend to be closer to comfort levels (level C), obtained psychoacoustically, exceeding these value in some cases, especially in basal electrodes ^13^.

As to etiology of deafness, we noticed that in progressive deafness cases, thresholds of NRT may be reduced ^14^. In the present study, we detected a slight increase in thresholds of telemetry in subjects with deafness for otosclerosis and meningitis (mean of 200CU and 192CU, respectively), whereas the mean of NRT threshold in cases of deafness by other etiologies was at about 188CU. In these two etiological processes there is pathological bone growth; in otosclerosis, caused by increase activity of osteoblasts (with deposit of bone matter), which peaks with increase in otic capsule, and in ossifying cochleopathy of meningitis, there is increase in otic capsule plus occlusion of tympanic, cochlear and vestibular scali, in most cases.

The bone that separates Rosenthal canal from the tympanic scala, if the thickness is increased (such as in the case of otosclerosis), could affect the current flow and provide more resistance to the passage of stimuli up to the ganglionar cells ^12^. At the same time, the excessive tissue and bone growth in the cochlea could affect the passage of electrical current, resulting in increase in levels of energy required for stimulation and, consequently, increase in auditory action potential ^11^.

The duration of deafness seemed to be determining in the thresholds of NRT.

I/O function in the amplitude growth curve inclination was greater in subjects who had had the deafness for less time. These functions would be closely related with the dynamic field and maximum levels of comfort (levels C) of the patient and the inclination of the curve could provide data about the number of surviving neurons of the spiral ganglion, which could help in the adjustment of the maps ^15^. Thus, we can infer that subjects with less time of deafness could have better cortical representation of the auditory memory for the growth function of loudness, and consequently, greater dynamic field than subjects with less time of auditory deprivation, which in turn, could affect more subjects with discomfort, not supporting high stimulation levels.

As to etiology of hearing loss, the variation of I/O function was small in the different pathologies of the studied group. Similarly, some authors reported that in sudden losses of hearing, I/O function should be greater than in progressive losses, observing a difference in values obtained by the inclination of the gain growth curves between patients with congenital deafness and post-lingual deafness, as well as a more marked inclination in case of children with pre-lingual deafness^3^, ^5^, ^14^.

Latency was the most stable characteristic among the studied one using telemetry system. Our findings are in agreement with the literature, in which latency of N1 should be between 0.2µs and 0.4µs with mean of 0.3µs^6^, ^14^. There is a description that the latency tends to be higher in basal electrodes than in apical electrodes, smaller in toddlers (between 12 and 24 months) than in adults and inversely proportional to amplitude. It is explained by the fact that less neural degeneration causes sensorial deprivation. Thus, the reduction of latency and increase in amplitude could be the result of the stimulation of large proportions of functional neurons of the spiral ganglion or large synchronous neural response ^3^.

Therefore, the variation of thresholds, the function of amplitude growth curve inclination (I/O) and the refractory properties of action potential responses of the 8th nerve could indicate differences in the neural population of the subjects, which may even reflect in the performance of auditory perception ^6^.

The advantages of performing NRT measures during the surgery go beyond the verification of system integrity. Many times the necessary levels for NRT detection exceed the comfort limits, especially in children. Intraoperative recording does not require this type of concern, given that the patient is sedated and the obtained values can be used from the first stimulation.

New research studies are required for further understanding about the characteristics and prevalence of responses obtained by NRT, as well as to determine their capacity to predict behavior thresholds so that we can use the procedure to program the electrodes.

Given that NRT reflex is only the activity of auditory pathway primary neurons, behavioral measurements are also necessary and important for the assessment of information processing throughout the whole pathway of the central nervous system ^16^.
